# Functional annotation and ENU

**DOI:** 10.1186/1756-0500-5-580

**Published:** 2012-10-24

**Authors:** Teresa M Gunn

**Affiliations:** 1 McLaughlin Research Institute, 1520 23rd St S, Great Falls, MT, 59405, USA

**Keywords:** Ethylnitrosourea, ENU, Mutagenesis, Mouse genetics, Functional annotation

## Abstract

Functional annotation of every gene in the mouse genome is a herculean task that requires a multifaceted approach. Many large-scale initiatives are contributing to this undertaking. The International Knockout Mouse Consortium (IKMC) plans to mutate every protein-coding gene, using a combination of gene trapping and gene targeting in embryonic stem cells. Many other groups are performing using the chemical mutagen ethylnitrosourea (ENU) or transpon-based systems to induce mutations, screening offspring for phenovariants and identifying the causative mutations. A recent paper in BMC Research Notes by Arnold *et al.* presents data from an ENU-based mutagenesis project that provides not only some of the first phenotype-genotype information for a large number of genes, but also a trove of information, all publicly available, that demonstrates the specificity and efficiency of ENU mutagenesis.

## Commentary

By the late 1990’s, sequencing of the human and mouse genomes was progressing rapidly and it was recognized that the next crucial (and ambitious) step would be to assign function to every gene [1-3]. In the laboratory mouse, the chemical mutagen ethylnitrosourea (ENU) emerged as a powerful and unbiased tool by which a large number of mutant alleles with phenotypic effects could be generated in a relatively short time-span, and one that had the power to create hypermorphic, hypomorphic and null alleles [reviewed in [4]. The main drawback to this forward genetic approach was in identifying the mutant genes: until fairly recently, this required mapping and positional cloning. Today, the use of second-generation sequencing platforms allows investigators to rapidly identify mutations by sequencing the exome or full genome of a small number of affected individuals. This should make ENU mutagenesis an even more attractive tool for future studies, but with other, reverse genetic projects underway, is ENU mutagenesis still a valuable approach?

Arnold *et al.*[5] provide data that helps answer this question. They present a compendium of 587 ENU-induced mutations in 519 genes, generated over a period of 10 years [5]. Of these, 185 mutations in 129 different genes caused a discernable phenotype, while 402 incidental mutations (i.e., not responsible for the phenotype under investigation) were identified in 390 genes. This large dataset allowed the authors to calculate the overall frequency at which ENU caused mutations, determine the frequency of different classes of mutations, and estimate how often different types of mutations cause phenovariance. They also analyzed which amino acid substitutions were more likely to be deleterious and which were more likely to be benign, and used their data to test the sensitivity and specificity of programs designed to assess the likelihood that a given amino acid substitution will result in loss of function of a protein.

One of the most valuable aspects of this work is that the data presented in this article have been made available using LabArchives, the laboratory notebook software, under a Creative Commons CC0 waiver. This means the authors have waived the copyright in their data, allowing other researchers to reuse these data without restrictions. BioMed Central has partnered with LabArchives to permanently link datasets and the peer-reviewed articles with which they are associated, with the goal to make these data openly available under terms that freely permit reuse [6,7]. The data and methods (including for the phenotypic screens they employed) associated with Arnold *et al.*’s work are freely available on the authors’ own website [8] as well as in a LabArchives Electronic Laboratory Notebook [9], which will remain available in perpetuity. Investigators can browse or search either site by gene name, chromosome, or phenotype. Mutation statistics are also posted at the Mutagenix website, with links to lists of mutants that fall into each category. Each phenovariant identified in the screen has its own entry that provides, among other things: background information on the gene; links to other alleles; a description (with photographs) of the mutant phenotype; a description of the ENU-induced mutation and its predicted effect on the protein; the putative mechanism by which the mutation disrupts gene/protein function; and what stock is available to interested investigators (for example, genomic DNA, sperm, frozen embryos or live mice). Most of the mutants generated in this screen are also openly available to other researchers, having been deposited to the Mutant Mouse Regional Resource Centers [10].

Other large-scale ENU mutagenesis centers have websites that can be searched by phenotype, but few, if any, of these other projects have identified the causative mutation for each one of their phenovariants. This greatly enhances the value of the Mutagenix mice as a resource because investigators can search for mutations that cause a certain phenotype or that disrupt a specific gene. With whole genome sequencing becoming evermore affordable, all ENU mutagenesis centers should be encouraged to identify all of the mutations in their mice. This would provide a resource to complement the one being produced by the International Knockout Mouse Consortium (IKMC), which is using genome sequence information with gene trapping and gene targeting to generate hypomorphic alleles for every protein-coding gene in the mouse [11]. If all available ENU-induced mutations were identified, researchers could search both resources for mutations targeting their genes of interest and have access to a wider variety of alleles. Since at least preliminary phenotypic analysis is performed on the ENU-induced mutants, investigators would have more information at their disposal when deciding whether one of these mutants would be useful to them, although it is likely that some of the mutants already analyzed have subtle phenotypes that have not yet been ascertained.

Will further ENU mutagenesis screens be fruitful? Despite the variety of mutants identified thus far, the genome does not appear to have been saturated by this approach. Not every gene has been targeted, and because ENU predominantly causes single base-pair substitutions, novel alleles with different effects on protein function can still be created in genes for which mutants already exist. A powerful use of ENU mutagenesis that has not been fully exploited is in the identification of modifiers of pre-existing mutants with known phenotypes. This approach can assign function to previously uncharacterized genes as well as new functions to characterized ones. It is also a powerful approach for identifying new components of disease pathways and potential therapeutic targets. As for large-scale ENU mutagenesis programs, a new and possibly fruitful direction to complement IKMC’s efforts would be mutagenesis of mouse embryonic stem cells (ESC), followed by whole-genome sequencing to identify mutations. If this data were made publicly available, investigators could search for mutations in their gene of interest and order the ESC to study in culture (as ESC or differentiated into a cell-type of interest) or to generate mutant mice. In a similar vein, the RIKEN BioResource Center has archived sperm from ~10,000 first generation (G1) mice generated in a large-scale mutagenesis project. Upon request, RIKEN will perform a PCR-based screen of their mouse mutant library (sperm and genomic DNA from G1 mice) to identify mutations in a gene of interest [12]. The analysis Arnold *et al*. present on the likelihood of a specific amino acid change to be deleterious will be particularly useful to help investigators predict whether a given mutation is likely to cause a phenotype. While ENU will not create conditional alleles (which remains one of the biggest strengths of the knockout mouse project), it can provide a wider variety of alleles (hyper- and hypomorphic). Successful functional annotation of the genome will require the combined power of both of these approaches.

## Competing interests

The author declares that she has no competing interests.
